# Report of Cases of Aneroticism in Women*For detailed method of treatment by simple operation, see March number of *Council*.

**Published:** 1896-06

**Authors:** Eugene P. Bernady

**Affiliations:** Philadelphia


### REPORT OF CASES OF ANEROTICISM IN WOMEN.*

BY EUGENE P. BERNADY, M. D., PHILADELPHIA.

Aneroticism in women, no doubt, may arise from numerous causes, but I believe that in many, if not the majority, of the cases, the cause lies in the condition of the clitoris and its cover.

But few physicians believe alike regarding the location of the sensitive area which causes sexual orgasm. Some will give the vagina, some the ovaries, while others the uterus; but in my judgment I believe undoubtedly the clitoris is the prime factor.

The series of cases reported below extends over a number of years, none under a year; therefore, the results can be more positively ascertained as regards their permanency.

CASE I. Young blonde, aged 18 years, married two years, mother of two children; first child still-born. It was during her lying-in that she complained of absence of sexual feeling. "What is that pleasure I hear so much about? I have never experienced it. The approaches of my husband I abhor." Within a week the husband came to my office with an attack of gonorrhea. While giving him a lecture he remarked: "I have

*For detailed method of treatment by simple operation, see March number of *Council*.

no pleasure with my wife. She is like a piece of marble." I determined to examine the clitoris of my patient; her condition made an easy excuse. I found the prepuce adherent to the glans, its margin red and irritated. Catching the margin of the prepuce with a pair of dressing forceps, I separated the adhesion. After washing the parts thoroughly with warm water, I smeared the glans with carbolized cosmoline. This treatment was kept up until the raw surfaces were completely healed. A year after, meeting her husband, he informed me that everything was satisfactory.

CASE II. Friend of the above, married five years, one child, complained of sexual apathy. Found the prepuce adherent, separated the adhesions, and pursued the same course of treatment as in Case I. Some months after I met the patient in the mountains spending her vacation. She informed me that orgasm was perfect.

CASES III., IV., and V. were women who had been married between a few months and two years. In all, the prepuces were adherent. The adhesions were carefully separated under cocaine. All the cases reported favorably. In Case V. the dissatisfaction was so great on the part of the husband, that, had it not been for the influence of the priest, he would have left his wife. Shortly after the operation he sent me his thanks with those of his wife.

CASE VI. Young woman, age 23 years; had been married about six months. The husband first consulted me, and stated that his wife since their marriage had never manifested any pleasure in the conjugal act. On examination the glans was completely bridged over by the labia minora. Under the influence of cocaine I slit the prepuce down about one-half inch, then thoroughly broke all the adhesions, and packed with iodoform gauze. The dressing was applied fresh every day, and in the course of ten days all the raw surfaces were healed. The result was entirely satisfactory to both. I have since confined the patient with two children.

CASES VII. and VIII. were women, married two years, who came to me for supposed uterine disease, an anxiety to know if they could have children, and lack of sexual pleasure. Examination of the uterus showed the organ normal; no tender ovaries; the prepuces were adherent. In Case VIII. hard balls of smegma could be felt under the prepuce. The adhesions were carefully separated, thoroughly washed with an antiseptic wash of biniodide of mercury, and then packed with iodoform gauze.

CASE IX. The husband came for treatment for acute gonorrhea. During treatment he informed me that he had been married five years, and that his wife had evinced so little pleasure at his approaches, that he was compelled to go where he was better treated. I examined his wife. She was a beautiful brunette, about 25 years of age; never had any children. She stated that she had never experienced any orgasm. Examination showed the prepuce adherent throughout. All adhesions were carefully separated, and treatment pursued as in the other cases. The result was perfect satisfaction to husband and wife.

These are only a few of the cases that I have operated on, and are a fair sample of the class of cases we have to deal with. The results have been, in my hands, invariably successful.—*The Medical Council.*

